# The Feasibility of Using Instagram Data to Predict Exercise Identity and Physical Activity Levels: Cross-sectional Observational Study

**DOI:** 10.2196/20954

**Published:** 2021-04-19

**Authors:** Sam Liu, Megan Perdew, Alexander Lithopoulos, Ryan E Rhodes

**Affiliations:** 1 School of Exercise Science, Physical and Health Education University of Victoria Victoria, BC Canada; 2 Department of Psychology University of Victoria Victoria, BC Canada

**Keywords:** social media, exercise identity, physical activity, physical fitness

## Abstract

**Background:**

Exercise identity is an important predictor for regular physical activity (PA). There is a lack of research on the potential mechanisms or antecedents of identity development. Theories of exercise identity have proposed that investment, commitment and self-referential (eg, I am an exerciser) statements, and social activation (comparison, support) may be crucial to identity development. Social media may be a potential mechanism to shape identity.

**Objective:**

The objectives of this study were to (1) explore whether participants were willing to share their Instagram data with researchers to predict their lifestyle behaviors; (2) examine whether PA-related Instagram uses (ie, the percentage of PA-related Instagram posts, fitness-related followings, and the number of likes received on PA-related posts) were positively associated with exercise identity; and (3) evaluate whether exercise identity mediates the relationship between PA-related Instagram use and weekly PA minutes.

**Methods:**

Participants (18-30 years old) were asked to complete a questionnaire to evaluate their current levels of exercise identity and PA. Participants’ Instagram data for the past 12 months before the completion of the questionnaire were extracted and analyzed with their permission. Instagram posts related to PA in the 12 months before their assessment, the number of likes received for each PA-related post, and verified fitness- or PA-related followings by the participants were extracted and analyzed. Pearson correlation analyses were used to evaluate the relationship among exercise identity, PA, and Instagram uses. We conducted mediation analyses using the PROCESS macro modeling tool to examine whether exercise identity mediated the relationship between Instagram use variables and PA. Descriptive statistical analyses were used to compare the number of willing participants versus those who were not willing to share their Instagram data.

**Results:**

Of the 76 participants recruited to participate, 54% (n=41) shared their Instagram data. The percentage of PA-related Instagram posts (*r*=0.38; *P*=.01) and fitness-related Instagram followings (*r*=0.39; *P*=.01) were significantly associated with exercise identity. The average number of “likes” received (*r*=0.05, *P*=.75) was not significantly associated with exercise identity. Exercise identity significantly influenced the relationship between Instagram usage metrics (ie, the percentage of PA-related Instagram posts [*P*=.01] and verified fitness-related Instagram accounts [*P*=.01]) and PA level. Exercise identity did not significantly influence the relationship between the average number of “likes” received for the PA-related Instagram posts and PA level.

**Conclusions:**

Our results suggest that an increase in PA-related Instagram posts and fitness-related followings were associated with a greater sense of exercise identity. Higher exercise identity led to higher PA levels. Exercise identity significantly influenced the relationship between PA-related Instagram posts (*P*=.01) and fitness-related followings on PA levels (*P*=.01). These results suggest that Instagram may influence a person’s exercise identity and PA levels. Future intervention studies are warranted.

## Introduction

Regular physical activity (PA) is critical to prevent chronic diseases and maintain overall well-being [1]. However, physical inactivity (ie, not meeting recommended guidelines) continues to be a major public health concern worldwide [2]. In Canada, approximately 79% of adults aged 18-39 did not meet the PA guidelines (150 minutes of moderate-to-vigorous PA per week) in 2015 [3]. Thus, there is an urgent need to find innovative solutions to better target and tailor PA interventions based on a person’s needs to maximize public health impact [4]. Much of the PA promotion research literature has been based in the social cognitive tradition, which is focused on increasing expectations about the benefits of PA, improving perceptions of capability, and developing goals and self-regulation tactics to enable behavior change [5-8]. The social cognitive–based behavior change theories suggest that intentions are proximal antecedents of behavior; thus, supporting positive PA intentions can lead to subsequent behavioral enactment [9]. This approach has shown some effectiveness in terms of behavior change, albeit with modest effects [5]. There is clearly a need to continue to investigate additional mechanisms that can assist in PA continuance.

One of these mechanisms may be identity. Identity represents a person’s self-categorization into a particular role [10]. This process assists in developing personal rules and standards of conduct with a particular behavior, such as PA [11]. As such, the central mechanism that sustains behavior becomes regulation around the identity standard [12]. Exercise identity refers to the degree to which an individual identifies himself/herself as an “exerciser” or someone who values and regularly engages in PA [13]. Discrepancies between the person’s exercise identity and the present situation (eg, being physically inactive) can create negative emotions, which are proposed to motivate the individual to close the gap between the present behavior and the identity standard [12,14]. In essence, identity is a reflexive self-regulating system of behavioral continuance/stability [10]. Exercise identity has emerged as an important construct for PA promotion because of the reciprocal relationship between role identities and behavior [15]. Self-report surveys, such as the Exercise Identity Scale, have been developed and rigorously tested for assessing an individual’s exercise identity, which has allowed for further investigation of the association between exercise identity and PA behavior [16].

Observational evidence has shown that PA behavior and identity have a reliable association in the medium to large effect size range [17]. Furthermore, the relationship is often independent of more traditional social–cognitive determinants (eg, intention) [17] and the mechanisms of identity dissonance and negative affect have been well-validated in hypothetical PA abstinence situations [18-20]. What has seen less attention, however, is the potential mechanisms or antecedents of identity development. Theories of exercise identity have proposed that investment, commitment and self-referential statements (eg, I am an exerciser), and social activation (comparison, support) may be crucial to identity development [13,21-23]. Another possible mechanism of identity is self-expression. Bem’s self-perception theory [24], for example, suggests that identity may be formed largely by observing one’s behavior and inferring self-categorization. This premise has some support in PA interventions from 2 small feasibility trials where participants who were encouraged to present PA-related imagery and self-expression (eg, a picture on the mantle) showed corresponding increases in identity [25,26]. However, the large focus on social media and self-expression across much of the population may be an even more effective mechanism of shaping identity.

Social media websites, such as Instagram, allow users to easily and freely communicate and express themselves by posting photos, status messages, or links that become instantly available to the public [27]. Infodemiology is a new area of study where researchers examine ways to use data generated on social media to better understand and inform public health problems in real time [28]. Infoveillance has referred to applications where infodemiology methods are utilized, particularly for surveillance purposes [29]. PA promotion interventions can potentially extract useful information from user-generated social data for infoveillance and to tailor health promotion efforts to improve PA levels. In fact, recent studies have shown the possibility of using social media and digital interventions to promote PA [30-32].

Researchers have already shown that behavior characteristics extracted from social media sites, such as Twitter, can be used to model alongside other biomedical data sets to inform PA on a population level [30-34]. We believe that it is critical to explore the use of social data from other platforms, such as Instagram, to expand the field of infodemiology. Instagram is a highly unique and user-friendly platform that allows users to share photos and videos on their mobile devices anywhere at any time. This platform has evolved into a visual-oriented culture, promoting photos first and text second. Instagram enables users to express their personalities and lifestyles with others and form relationships with users that share similar interests and values. In contrast to Twitter and Facebook, Instagram’s “photo-first” culture may contribute to different user behavior and motivation [35]. Approximately 71% of online adults in America have an Instagram account [36] compared with 38% of adults (18-29 years of age) that use Twitter [37]. In Canada, 37% of adults have a social networking account, and among adults aged 18-34, about 65% have an Instagram account [38]. An Instagram study suggested that analysis of the content of Instagram pictures, color analysis (amount of saturation brightness, photo filters), frequency of posts, patterns of followings, algorithmic face detection, and the number of likes are associated with symptoms of depression [39].

**Figure 1:**
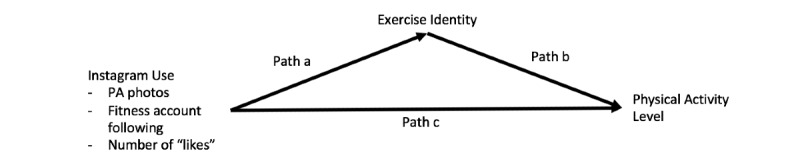
Hypothesized mediation model including Instagram use, exercise identity, and physical activity level.

However, it remains unclear whether PA-related markers extracted from Instagram are associated with a person’s exercise identity and subsequent PA level. Furthermore, Twitter data are designed to be publicly available, whereas Instagram data are not publicly available by default [40]. Users may perceive their Instagram data as being more private and sensitive [41]. The percentage of participants who are willing to share their Instagram data for health behavior monitoring is currently unclear. Thus, the objectives of this study were to (1) explore whether participants were willing to share their Instagram data with researchers to predict their lifestyle behaviors; (2) examine whether PA-related Instagram uses (ie, the percentage of PA-related Instagram posts, fitness-related followings, and the number of likes received on PA-related posts) were positively associated with exercise identity; and (3) evaluate whether exercise identity mediates the relationship between PA-related Instagram use and weekly PA minutes. Commensurate with self-perception theory [24] and past research [25,26], we hypothesized (1) more than 50% of the participants would be willing to share their Instagram data for health infoveillance, (2) PA-related Instagram use was positively associated with exercise identity, and (3) exercise identity would mediate the relationship between PA-related Instagram use (ie, number of likes) and PA minutes. Figure 1 presents a visual representation of the hypothesized mediation model.

## Methods

### Participants and Procedure

This cross-sectional study recruited university students (n=76) from the University of Victoria, British Columbia, Canada, using research posters and online advertisements. The rolling recruitment strategy took place between June 30, 2018, and July 1, 2019. Inclusion criteria required that participants be Instagram users, and between the ages of 18 and 29. Exclusion criteria included the inability to comprehend English. Interested participants were asked to contact the research team. The research team obtained consent from all eligible participants prior to data collection. During the consent process, participants were notified of the study objectives and that the sharing of their Instagram data was voluntary. All data obtained in connection with the study remained confidential. Confidentiality was maintained by means of encrypting on a secure server. All survey data and ID numbers were used on all databases. Researchers had access to participant Instagram data for up to 1 year from the start of the study.

Eligible participants who gave consent to participate in the study were asked to complete a questionnaire to record their demographic characteristics, daily Instagram use duration, and evaluate their current exercise identity and PA levels. Participants were also asked if they are willing to share their Instagram data. Participants that refused to share their Instagram data were given the option to write down reasons for not sharing. Those participants who agreed to share their Instagram data were asked to follow an Instagram research account in order for researchers to extract their Instagram data (photos, likes, followings). Researchers analyzed the previous 12 months of Instagram data from the completion date of the exercise identity and PA questionnaires. This study received research ethics approval from the research ethics board at the University of Victoria (protocol number: 17-294).

### Measures

Demographic information (age, sex, and ethnicity) and daily Instagram usage time were collected using self-report instruments. PA levels were assessed using the Recent Physical Activity Questionnaire (RPAQ) [42]. Minutes of PA at low (metabolic equivalent of task [MET] < 6/week), moderate (MET 3-6/week), and high (MET > 6/week) intensities were calculated based on the RPAQ scoring instruction [42].

Exercise identity was assessed using the Exercise Identity Scale [16], which is a 9-item questionnaire on a 5-point Likert scale from 1 (strongly disagree) to 5 (strongly agree) (α=.92). Participants’ exercise identities were determined by calculating a total score from the 9-item questionnaire; thus, scores ranged from 9 to 45 whereby a higher score represented a stronger exercise identity.

Participants’ Instagram data were extracted using a customized program [43], which enabled the researchers to export users’ posts, likes received for each post, and followings into separate Microsoft Excel files. Instagram posts extracted during the past 12 months before the completion of the study questionnaire were manually coded by 2 trained research assistants using standardized criteria to determine whether posts were PA related or not (eg, binary coded). PA-related posts were defined as those posts suggesting participants performed physical activities defined by the guidelines for exercise testing published by the American College of Sports Medicine [44]. Specifically, Instagram picture and video posts were identified as PA related if they included the person in sporting attire or the person in a location to perform PA (eg, kayaking, skiing, swimsuit, hiking) individually or in a group setting. Any discrepancies between the coders were resolved through discussion. The percentage of the number of PA-related posts relative to the total number of Instagram posts was calculated for each participant. The average number of likes for PA-related posts for each participant was also calculated.

An analysis of participants’ Instagram “followings” was completed to identify the number of verified fitness or exercise-related Instagram accounts each participant followed. The verified fitness- or exercise-related Instagram accounts means Instagram has confirmed that an account is the authentic presence of the public figure, celebrity, or global brand it represents. A list of the most popular 300 verified fitness- or exercise-related Instagram accounts was compiled from publicly available Instagram rankings [45]. The percentage of exercise-related Instagram accounts following relative to the total number of Instagram followings was computed for each participant.

### Statistical Analysis

All analyses were performed using the standard SPSS version 26.0 for Mac (SPSS, Inc./IBM), with a significance level set at *P*<.05. Descriptive statistical analyses were used to compare the number of willing participants with those who were not willing to share their Instagram data. Participants that were not willing to share their Instagram data were asked to provide reasons for not sharing in an open-ended question. Responses from these questions were grouped to create common themes. Independent *t* tests were used to compare differences between those groups (Instagram data shared versus Instagram data not shared) on demographics, PA, and exercise identity. A chi-square test was used to compare differences between categorical variables.

We examined normality (eg, skewness > 2 and kurtosis > 3) of all variables to determine whether any transformations were required by conversion to z-scores [46]. Pearson correlation analyses were used to evaluate the relationship among exercise identity, PA, and Instagram use metrics (percentage of PA-related Instagram post, percentage of fitness-related Instagram followings, and average number of “likes” on PA-related posts). Mediation analyses were based on 5000 bootstrapped samples using Hayes PROCESS Macro version 3.5 [47]. Multiple mediation analyses was used to examine whether exercise identity mediated the relationship between Instagram use variables and PA level [47]. This process involved examining path a, the association between Instagram use (independent variable) and exercise identity; path b, the impact of exercise identity (mediator variable) on PA level; and path c, the effect of Instagram use on PA levels (outcome variable). All analyses were controlled for age and sex. The 95% CIs must not cross 0 to satisfy the criteria for mediation.

## Results

### Participant Characteristics

A total of 76 participants were recruited and completed the study. Participant characteristics are presented in Table 1. The mean age was 19.7 (SD 0.31) years and the sample consisted of 72% female (55/76). Overall, 46% (35/76) of participants did not agree to share their Instagram data. Of these participants, 14 participants provided reasons for not sharing. Privacy was a major concern; specifically, there were concerns with (1) data oversight to prevent misusage of data and technology (n=9), (2) limitations to remain anonymous using Instagram photos (n=4), and (3) data ownership (n=1). Participants that were not willing to share their Instagram data reported significantly fewer weekly PA minutes at moderate to high intensity (MET ≥3/week) relative to participants that agreed to share their Instagram data (*P*=.04); however, there were no significant differences between groups for demographic characteristics, exercise identity, weekly PA minutes at low intensity (MET <3/week), and daily Instagram usage (Table 1).

A total of 41 participants agreed to share their Instagram data. The mean number of posts during the 12-month study period was 29.6 (SD 4.7). Examination of shared Instagram data revealed that, on average 7.3 (SD 1.1) or 25% of Instagram posts shared during the previous 12 months were related to PA. The mean number of Instagram following per person was 439.3 (SD 258) accounts. The mean number of verified PA- or fitness-related Instagram accounts following per user was 3 (SD 3.14). The mean number of “likes” received on all posts was 102.2 (SD 72.4) and the mean number of “likes” received on PA-related Instagram posts was 99 (SD 73).

**Table 1:** Baseline characteristics.

Characteristics			Instagram data shared (n=41)		Instagram data not shared (n=35)		*P* value
Age, mean (SD)			19.66 (2.08)		20.03 (1.81)		.42
Sex (female), n (%)			27 (66)		28 (80)		.20
**Ethnicity, n (%)**							.70
	White	29 (71)		24 (69)			
	East Asian	6 (15)		6 (17)			
	Black	0 (0)		1 (3)			
	Other (mixed, Spanish, South Asian)	6 (15)		4 (11)			
Exercise identity, mean (SD)			34.00 (8.20)		34.79 (7.16)		.67
Weekly PA^a^ minutes at low intensity (MET^b^ <3/week), mean (SD)			38.51 (135.94)		22.92 (59.94)		.50
Weekly PA min at moderate to high intensity (MET ≥3/week), mean (SD)			476.37 (542.40)		263.82 (238.27)		.04
Instagram usage (hours/day), mean (SD)			1.87 (1.04)		2.32 (1.91)		.29

^a^PA: physical activity.

^b^MET: metabolic equivalent of task.

### The Relationship Among Exercise Identity, Physical Activity, and Instagram Use Metrics

Normality analysis revealed that weekly PA minutes at moderate to high intensity (MET ≥3/week) and fitness-related Instagram followings percentage were kurtotic (ie, values ≥3). These variables were transformed by conversion to z-scores. The Pearson correlation analyses are shown in Table 2. The results indicated that the percentage of PA-related Instagram posts (*r*=0.38; *P*=.01) and fitness-related Instagram (*r*=0.39; *P*=.01) followings were significantly associated with exercise identity. The strengths of the associates were moderate. We did not find that the average number of “likes” received (*r*=0.05, *P*=.75) to be significantly associated with exercise identity.

**Table 2:** Correlation analyses among exercise identity, physical activity, and Instagram use metrics.

Metrics			Variables								
			1		2		3		4		5
**Pearson *r***											
	Exercise identity	—									
	Weekly PA^a^ minutes at moderate to high intensity (MET^b^ ≥3/week)	0.49		—							
	Percentage of physical activity–related Instagram posts	0.38		0.03		—					
	Percentage of verified fitness-related Instagram following	0.39		0.23		0.19		—			
	Average number of “likes” received for the physical activity–related Instagram posts	0.05		0.01		–0.04		–0.13		—	
***P* value**											
	Exercise identity	—									
	Weekly PA^a^ minutes at moderate to high intensity (MET^b^ ≥3/week)	.001		—							
	Percentage of physical activity–related Instagram posts	.01		.86		—					
	Percentage of verified fitness-related Instagram following	.01		.14		.23		—			
	Average number of “likes” received for the physical activity–related Instagram posts	.75		.95		.78		.43		—	

^a^PA: physical activity.

^b^MET: metabolic equivalent of task.

### Mediation Analysis: Exercise Identity, Percentage of Physical Activity–Related Instagram Post, and Physical Activity

Exercise identity significantly influenced the relationship between the percentage of PA-related Instagram posts and PA levels (indirect effect: 3.28, 95% CI 0.54 to 8.11; direct effect: –3.65, 95% CI –9.70 to 2.39). As indicated in Table 3, a unit increase in percentage of PA-related Instagram posts was associated with a 0.10-unit increase in exercise identity; a unit increase in exercise identity was associated with 34 minutes of PA (MET ≥3/week) increase. Approximately 29% of the variance was accounted for by the predictors (*R*^2^=0.29).

**Table 3:** Mediation analyses of the effects of exercise identity on Instagram use and physical activity levels.a

Effects		B	95% CI	β	*P* value
**Instagram PA^b^photos—PA levels (MET^c^** ≥**3/week)^d^**					
	Path a: Instagram PA photos—exercise identity	0.10	0.01 to 0.18	.33	.02
	Path b: Exercise identity—PA levels	34.0	11.66 to 56.36	.51	<.01
	Path c: Instagram PA photos—PA levels	–3.65	–9.70 to 2.39	–.18	.22
**Instagram PA following—PA levels (MET** ≥**3/week)^e^**					
	Path a: Instagram PA following—exercise identity	3.18	1.02 to 5.33	.48	<.001
	Path b: Exercise identity—PA levels	29.34	7.16 to 51.52	.44	.01
	Path c: Instagram PA following—PA levels	32.75	–129.17 to 194.68	.06	.68
**Instagram PA “likes”—PA levels (MET** ≥**3/week)^f^**					
	Path a: Instagram PA “likes”—exercise identity	0.02	–0.01 to 0.06	.21	.17
	Path b: Exercise identity—PA levels	28.6	6.75 to 50.47	.43	.01
	Path c: Instagram PA “likes”—PA levels	0.28	–2.06 to 2.61	.04	.81

^a^Model covariates include age, gender, B (unstandardized beta), and β (standardized beta).

^b^PA: physical activity.

^c^MET: metabolic equivalent of task.

^d^Indirect effect: 3.28 (95% CI 0.54 to 8.11); direct effect: –3.65 (95% CI –9.70 to 2.39).

^e^Indirect effect: 91.9 (95% CI 36.93 to 174.82); direct effect: 60.53 (95% CI –115.15 to 236.21).

^f^Indirect effect: 0.69 (95% CI –0.10 to 2.28); direct effect: 0.28 (95% CI –2.06 to 2.61).

### Mediation Analysis: Exercise Identity, Fitness-Related Instagram Followings, and Physical Activity

Exercise identity significantly influenced the relationship between the percentage of verified fitness-related Instagram following and PA level (MET ≥3/week; indirect effect: 91.9, 95% CI 36.93 to 174.82; direct effect: 60.53, 95% CI –115.15 to 236.21). A unit increase in percentage of fitness-related Instagram following leads to a 3.18-unit increase in exercise identity; a unit increase in exercise identity was associated with an increase of 29.34 minutes of PA (MET ≥3/week; Table 3). Approximately 26% of the variance was accounted for by the predictors (*R*^2^=0.26).

### Mediation Analysis: Exercise Identity, Physical Activity–Related Instagram “Likes,” and Physical Activity

Exercise identity did not significantly influence the relationship between the average number of “likes” received for the PA-related Instagram posts and PA level (MET ≥3/week; indirect effect: 0.69, 95% CI –0.10 to 2.28). We did not observe a significant direct effect between the number of “likes” received on PA-related Instagram posts and PA minutes (direct effect: 0.28, 95% CI –2.06 to 2.61; Table 3).

## Discussion

### Principal Findings

The objectives of this study were to explore whether participants were willing to share their Instagram data with researchers to predict their lifestyle behaviors and examine the relationship among PA-related Instagram metrics, exercise identity, and PA. We found that the majority of participants were willing to share their Instagram data for monitoring lifestyle behaviors. We also found that only PA-related Instagram posts and fitness-related followings were significantly associated with an increase in exercise identity. Furthermore, we found that exercise identity significantly influenced the relationship between certain Instagram use metrics (eg, PA-related Instagram posts and fitness-related followings) and PA level. To our knowledge, this is one of the first studies to examine the relationship among Instagram use, exercise identity, and PA levels. The methods and the findings from this study can be used to inform future infodemiology PA-related studies.

Our results suggest that over 50% (41/76, 54%) of the users were willing to share their Instagram data for monitoring their PA and health-related behaviors. This observation was supported by our hypothesis. It was worth noting that participants that agreed to share their data reported significantly higher weekly PA minutes at moderate to high intensity (MET ≥3/week) compared with participants who were not willing to share their data. A potential explanation is that those who were less active felt guilty for not showing consistent behavior with their social media posts. Individuals may not always portray their real selves on social media sites due to factors such as lower self-esteem, self-reflection, and self-concept clarity [48]. Alternatively, those participants may feel being monitored or judged for what they were posting, given the purpose of this study [27,49]. Concerns over privacy were the main reasons for not sharing their Instagram data. Unlike Twitter data, Instagram data are not publicly available by default, and the users mainly share pictures instead of text. Thus, this poses a limitation to remain anonymous when analyzing Instagram photos. Our findings reinforced the ethical challenges faced in using social data for public health monitoring. There is a need to establish “best-practice” standards for analyzing social media data while simultaneously acknowledging peoples’ individual rights and respecting their privacy [50,51]. Previous studies have shown that users are more likely to accept the use of social media data for research if there is an oversight body that protects user data to ensure that their data are not being used beyond the intended purpose and that user data can remain anonymous to protect the identity of the user [52,53]. Overall, these privacy considerations need to be addressed for the future development of social data monitoring technology.

Based on the self-perception theory [24] and past research [25,26], we hypothesized that PA-related Instagram use would be positively associated with exercise identity and that exercise identity would mediate the relationship between PA-related Instagram use and PA minutes. Interestingly, we observed that only the percentage of PA-related Instagram posts and fitness-related followings were positively associated with exercise identity. Exercise identity also significantly influenced the relationship between these Instagram usage metrics and PA level (MET ≥3/week). Exercise identity did not influence the relationship between the number of “likes” and PA level. Previous research has demonstrated that social media “likes” can contribute to PA engagement [54,55]. The Instagram “likes” metric is unlike the number of followings and posts metrics because users do not have direct control over the number of “likes” received. The lack of consistency of our findings compared with previous literature may be attributed to the way individuals are using their Instagram in our sample. “Surveillance users” (eg, users focused on monitoring other users) may post lesser often and have a lesser number of followers, leading to a lower number of “likes” compared with “cool-ness users” who are motivated to become popular [56,57]. Thus, the types of users need to be taken into consideration for future studies.

Findings from this study have several implications for PA promotion research. First, Instagram may have the potential to target identity antecedents through the reception of both inspirational and informative posts about exercise from respected verified fitness accounts (ie, enhancing self-efficacy and congruency with the self) [58-60]. Based on the self-perception theory [24] and self-definition model [21], future PA promotion interventions using Instagram may target personal investment, perceived commitment (ie, engaging with relevant fitness groups/users), social activation (ie, contributing and receiving support from fitness groups/users), and self-expression (ie, share photos of their PA or fitness-related experiences or write about these activities using social media), as these may play a role in strengthening an individual’s exercise identity and subsequent PA behaviors [22,24]. However, it is worth noting that Instagram as a medium for expressing one’s PA behavior with the end goal being to strengthen individuals’ exercise identities may only be appealing to certain segments of the population. Second, results from this study extend previous Infodemiology research that Instagram data may be used to provide insights into health-related outcomes [39]. Future studies can build upon the methods used in this study and may also explore other Instagram metrics (eg, frequency of post) in PA infodemiology studies. Finally, PA researchers can leverage these social media analysis techniques to build prediction tools to monitor PA on a population level in real time. These tools may in the future aid public health agencies in identifying particular PA-related trends in various geographical areas on which to focus health and wellness initiatives.

There are potential negative aspects associated with using Instagram for the purpose of promoting PA and forming or strengthening one’s exercise identity that must be considered. For example, recent movements known as “fitspiration” and “fitspro” have become popular on Instagram to inspire and motivate others to eat a healthy diet and exercise regularly. Fitspiration provides many positive attributes, such as accountability for exercising and education about fitness [61]. However, some women sharing fitspiration posts on Instagram may indicate disordered eating and exercise behaviors [62]. Despite the potential of using Instagram to motivate adherence to a healthy lifestyle, it is important for future interventions to consider the potential negative effect of social media on mental and physical health. Future studies need to examine ways to create a positive environment where participants feel comfortable posting pictures of their exercise experience and journey to fitness.

In terms of the limitations of the study, our sample only included university students which may limit the ability to generalize our findings beyond our sample. Future studies should examine the relationship between social media posts and PA-related behavior in middle-aged and older adults. Another limitation is that we used a list of verified fitness-related Instagram account in our analysis. It is possible that users may follow fitness-related Instagram accounts that are not verified and did not make it onto our list. Thus, this means that the observed relationship between exercise identity and the percentage of the fitness-related Instagram following needs to be interpreted with caution. Self-report questionnaires may result in confirmation bias. Moreover, it may be possible that some users’ may have deleted photos, and thus affecting the number of PA-related photos in our analysis. Finally, the lack of longitudinal data for exercise identity and PA levels was another limitation when conducting mediation analysis. Thus, the mediation analysis result needs to be interpreted with caution. Future study with longitudinal data is needed to build more complex multivariate models to better examine mediation effects.

### Conclusion

This study examined the relationships among Instagram use, exercise identity, and PA levels. Findings from this study demonstrated that over 50% (41/76, 54%) of participants were willing to share their Instagram data with researchers for the purpose of monitoring and predicting PA behaviors. Exercise identity significantly influenced the relationship between Instagram use (eg, PA-related Instagram posts and fitness-related followings) and PA level. Results from this study suggest that there is an association between Instagram posts and PA-related outcomes (ie, exercise identity and PA levels). Future studies should examine whether Instagram may be used for a future intervention to help form or strengthen an individual’s exercise identity, particularly if the individual can be supported by their “social world.”

## Abbreviations

MET: metabolic equivalent of task
PA: physical activity
RPAQ: Recent Physical Activity Questionnaire
